# Absorbable versus Nonabsorbable Sutures for Facial Skin Closure: A Systematic Review and Meta-analysis of Clinical and Aesthetic Outcomes

**DOI:** 10.1055/a-2318-1287

**Published:** 2024-06-19

**Authors:** Kashish Malhotra, Sophie Bondje, Alexandros Sklavounos, Hatan Mortada, Ankur Khajuria

**Affiliations:** 1 Department of Surgery, Dayanand Medical College and Hospital, Ludhiana, Punjab, India; 2 Department of ENT Surgery & Cancer Services, Torbay Hospital, Torquay, United Kingdom; 3 Urology Division, Department of Surgery, Addenbrooke's Hospital, University of Cambridge, United Kingdom; 4 Division of Plastic Surgery, Department of Surgery, King Saud University Medical City, King Saud University, Riyadh, Saudi Arabia; 5 Department of Plastic Surgery and Burn Unit, King Saud Medical City, Riyadh, Saudi Arabia; 6 Department of Surgery & Cancer, Imperial College London, London, United Kingdom; 7 Institute of Applied Health Research, University of Birmingham, Birmingham, United Kingdom

**Keywords:** absorbable, nonabsorbable, facial wounds, suture material, patient-reported outcomes, skin closure, satisfaction

## Abstract

When repairing facial wounds, it is crucial to possess a thorough understanding of suitable suture materials and their evidence base. The absence of high-quality and comprehensive systematic reviews poses challenges in making informed decisions. In this study, we conducted a review of the existing literature and assessed the quality of the current evidence pertaining to the clinical, aesthetic, and patient-reported outcomes associated with absorbable and nonabsorbable sutures for facial skin closure.

The study was registered on Prospective Register of Systematic Reviews. We conducted searches on Embase, Ovid, and PubMed/MEDLINE databases. Only randomized controlled trials (RCTs) were eligible for inclusion in this study. Additionally, the risk of bias in the randomized studies was assessed using Cochrane's Risk of Bias Tool.

The study included a total of nine RCTs involving 804 participants with facial injuries. Among these injuries, absorbable sutures were utilized in 50.2% (403 injuries), while nonabsorbable sutures were employed in 49.8% (401 injuries). The analysis of cosmesis scales revealed no statistically significant difference between absorbable and nonabsorbable sutures regarding infections ( *p* = 0.72), visual analog scale ( *p* = 0.69), wound dehiscence ( *p* = 0.08), and scarring ( *p* = 0.46). The quality of the included studies was determined to have a low risk of bias.

Absorbable sutures can be considered a suitable alternative to nonabsorbable sutures, as they demonstrate comparable aesthetic and clinical outcomes. Future high-quality studies with a level I evidence design and cost-effectiveness analysis are necessary to enhance clinician–patient shared decision-making and optimize the selection of suture materials.

Level of evidence is I, risk/prognostic study.

## Introduction

Surgical wound healing is influenced by multiple factors, including patient characteristics, wound characteristics, and technical factors. 1 When performing surgeries, especially those involving facial injuries, it is crucial to consider cosmesis as an important outcome. 2 Surgeons are often evaluated based on their ability to create fine, linear, and inconspicuous scars to enhance the cosmetic appearance. 2 To achieve this goal, it is essential to have a basic understanding of suture materials and the appropriate techniques for their use.

Sutures can be classified based on various characteristics, such as absorbability, material composition (natural or synthetic), and structure (monofilament or multifilament). 3 Nonabsorbable sutures, such as nylon and polypropylene, are known for their resistance to degradation by living tissues, while natural materials like silk, linen, cotton, and surgical steel are also used. 3 Nonabsorbable sutures are often preferred for their strength and minimal inflammatory response. However, multifilament sutures have a higher risk of harboring bacteria and causing infection compared with monofilament sutures, which pass through tissues more easily. 4 Additionally, nonabsorbable sutures can cut tissues when tied under tension, and their high suture memory can make them challenging to handle and maintain knot security. 5

In contrast, absorbable sutures are designed to be absorbed by the body over time, depending on the specific material and brand used, with minimal tissue reaction. Examples of absorbable sutures include natural surgical gut, polyglactin (vicryl), polyglycolic acid (dexon), glycolic acid (Maxon), and polydioxanone (PDS). 5 Absorbable sutures are often braided to simplify knot tying and handling. One advantage of absorbable sutures is that they do not require removal, which can reduce patient anxiety, particularly in cases involving children and the elderly. 3 6

Several studies have demonstrated that absorbable sutures are cost-effective and do not compromise cosmetic outcomes. 7 8 However, some studies have cautioned against their use for skin closure due to the potential formation of “railroad track” scars, which can occur depending on the closure technique employed. 9 Cost savings can be achieved by using a single suture pack for both skin layers, eliminating the need for a second clinic appointment or additional procedures for suture removal under sedation or anesthesia, particularly in pediatric cases. 10 Despite the extensive debates surrounding sutures in various specialties, there is a need for high-quality research that delves deeper into the topic, going beyond general surgical suture techniques and material choices.

When it comes to facial wounds, closure methods are of utmost importance to patients due to the significant impact on cosmetic outcomes, and there is a considerable psychological burden and stigma associated with facial wounds. 8 Ideally, wound closure methods for facial injuries should be time-efficient, easy to perform, cost-effective, and result in optimal cosmetic outcomes. 10 The aim of this systematic review and meta-analysis is to evaluate the available literature and compare the clinical, aesthetic, and patient-reported outcomes associated with absorbable and nonabsorbable sutures for facial skin closure.

## Methods and Materials

### Registration

This systematic review adhered to the Preferred Reporting Items for Systematic Reviews and Meta-Analyses (PRISMA) guidelines, which provide a standardized framework for conducting and reporting systematic reviews. 11 Additionally, the review followed the Cochrane review methods, which are widely recognized as a gold standard in systematic reviews. 12 The study protocol was registered in advance in the National Institute of Health Research Prospective Register of Systematic Reviews (PROSPERO) under the registration number CRD42021267037. 13 By registering the protocol in PROSPERO, the study aims to enhance transparency and reduce the risk of bias in the review process.

### Search Strategies

The systematic search was conducted using multiple databases, including MEDLINE (OVID SP), Embase (OVID SP), PubMed, Cochrane Controlled Register of Trials (CENTRAL), and Web of Science. The search was not limited by language or geographical restrictions to ensure comprehensive coverage. The search strategy was developed through a combination of Medical Subject Heading terms, free-text keywords, and Boolean logical operators, in consultation with the senior authors. Additionally, the references' lists of the included articles were screened to identify any relevant references. Below is an example of a search strategy used for Embase (OVID SP), which was adapted for other databases:

Example search strategy for Embase (OVID SP):

1. “facial wound” or “exp face injury” (69514)
2. “exp wound closure” or “exp laceration” (50526)
3. Combine the results of step 1 and step 2 (1425)
4. “exp absorbable suture” (11784)
5. “exp nonabsorbable suture” or “absorbable” or “nonabsorbable” or “non absorbable” (12715)
6. Combine the results of step 4 and step 5 (2046)
7. “exp patient-reported outcome” or “exp treatment outcome” (2101643)
8. “exp satisfaction” or “exp patient satisfaction” (282202)
9. Combine the results of step 7 and step 8 (61279)

### Screening and Selection of Studies

All studies were extracted following a database search and populated into an Excel spreadsheet. First, two researchers, K.M. and S.B., independently screened the titles and abstracts. In case of any discrepancies, the article proceeded to a full-text review. Thereafter, the full texts of the included studies were independently screened for eligibility by K.M. and S.B. Any discrepancies were discussed and resolved through mutual consensus, and the senior authors, A.S. and A.K., were consulted for the final determination of the article's inclusion or exclusion. Data extraction from the full-text articles was performed using a standardized extraction form by K.M. and S.B. Any discrepancies were resolved through consensus or with referral to the senior authors, A.S. and A.K. The following data were extracted from each study: first author, year of publication, sample size, suture types, follow-up duration, country of patients, gender, validated questionnaire responses, and reported complications.

The primary outcome of this systematic review was to examine the levels of clinical complications associated with absorbable and nonabsorbable sutures. Specifically, the review assessed complications such as erythema, infections, wound dehiscence, the presence or absence of stitch marks, and the frequency of keloid/hypertrophic scars. Additionally, the review analyzed patient-reported outcomes in terms of cosmetic, functional, and symptomatic domains. To assess these outcomes, the review considered the use of various validated questionnaires, including the visual analog scale (VAS), visual Aanalog cosmesis scale (VACS), and other relevant measures. The objective was to identify any differences in outcomes between the use of absorbable versus nonabsorbable sutures.

### Study Design and Criteria

The systematic review focused on primary human studies that examined and compared the differences in clinical, aesthetic, and patient-reported outcomes between absorbable and nonabsorbable sutures for facial skin closure. The inclusion and exclusion criteria for the review were as follows:

#### Inclusion Criteria

1. Clinical studies: Only randomized controlled trials (RCTs) were considered to ensure the highest level of evidence synthesis.
2. Patients requiring facial skin closure via suturing: The studies included patients who underwent suturing for facial skin closure, without any specific restrictions based on age, ethnicity, or other health status factors.

#### Exclusion Criteria

1. Duplicates: Duplicate studies were excluded to avoid redundancy.
2. Case reports: Individual case reports were not included in the review.
3. Conference abstracts: Studies presented as abstracts at conferences were excluded.
4. Simulation studies: Studies focusing on simulation rather than actual patient outcomes were not included.
5. Case series: Studies reporting on a series of cases without a control group were excluded.
6. Review articles: Reviews summarizing existing evidence were not considered primary studies.
7. Molecular studies: Studies focusing on molecular aspects without outcome measurement were excluded.
8. Original reports other than RCTs: Studies that did not meet the criterion of being an RCT were excluded.
9. Technical descriptions without outcome measurement: Studies solely describing technical aspects without reporting outcomes were not included.
10. Clinical studies in nonhuman subjects: Studies conducted on nonhuman subjects were excluded from the review.

By applying these criteria, the systematic review aimed to include only RCTs that focused on absorbable and nonabsorbable sutures for facial skin closure and reported relevant clinical, aesthetic, and patient-reported outcomes in human subjects.

### Risk of Bias and Quality Assessment

The quality of the included RCTs was assessed using the Cochrane Risk for Bias Tool. 14 This tool consists of various domains, and judgments within each domain were used to determine an overall risk of bias judgment across five main domains. These domains focus on trial design, conduct, and reporting, and utilize specific questions to gather information relevant to the risk of bias. An algorithm was applied to these judgments, resulting in assessments of “low” risk of bias (indicating low risk across all domains), “some concerns” (indicating some concerns in at least one domain), or “high” risk of bias (indicating high risk or some concerns in multiple domains). The assessment of risk of bias was performed independently by two authors (K.M., S.B.), and any disagreements were resolved through consensus after consulting with the senior authors (A.S., A.K.). Furthermore, the articles included in the review were evaluated according to the level of evidence and grading recommendations of the American Society of Plastic Surgeons. 15 This evaluation provided additional insights into the strength of the evidence presented in the articles. By employing these assessment tools and guidelines, the systematic review aimed to ensure a comprehensive evaluation of the quality and level of evidence of the included studies.

### Statistical Analysis

The statistical analysis for this systematic review was performed using Review Manager (RevMan) Version 5.4, which is a software developed by The Nordic Cochrane Center and The Cochrane Collaboration in Copenhagen (2020). For continuous variables, such as evaluation scales, the mean difference was calculated as the measure of effect. RevMan was used as the data analysis tool to perform this calculation. Regarding categorical variables, such as complication rates, the outcomes were recorded as the number of events with the total number of patients in each group. The relative risk (RR) with a 95% confidence interval (CI) was calculated using RevMan to analyze these outcomes.

The total number of patients in each group was collected for the outcome parameters, and a quantitative assessment was conducted based on clinical, aesthetic, and patient-reported outcomes. The meta-analysis was performed using a random effects model, which takes into account the variability between studies. In cases of low heterogeneity, a fixed effect model was used, assuming a consistent effect size across all studies and minimizing the impact of between-study variability. The results of the meta-analysis were expressed as the mean difference for continuous variables or the RR with a 95% CI for categorical variables. Funnel plots were used to evaluate publication bias in the meta-analysis. A significance level of *p* < 0.05 was considered statistically significant, indicating that the observed results were unlikely to have occurred by chance.

## Results

### Primary Search Results

**Fig. 1:**
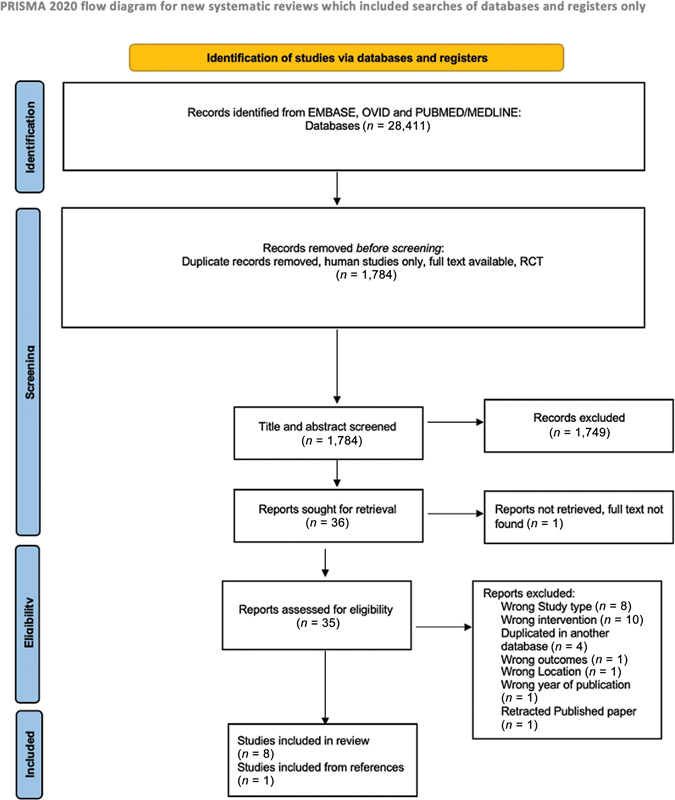
The flowchart of the reviewed studies according to PRISMA. PRISMA, Preferred Reporting Items for Systematic Reviews and Meta-Analyses; RCT, randomized controlled trial.

Our initial search of three main databases, namely Embase, Ovid, and PubMed/MEDLINE, yielded a total of 28,411 papers. After applying our exclusion criteria, 26,627 papers were deemed irrelevant and were excluded from further analysis. The remaining 1,784 papers underwent screening by reading titles and abstracts. From this screening process, 1,749 papers were excluded as they did not meet the inclusion criteria. A total of 35 full-text papers were retrieved and assessed for eligibility. Among these, nine RCT studies published between 2003 and 2020 were included in our review. 16 17 18 19 20 21 22 23 24 Twenty-three papers were excluded from the review due to being of the wrong study type, intervention, location, or duplication. Additionally, one additional paper was identified through reference screening. The majority of the included studies ( *n* = 6) were conducted in the United States, while two studies were conducted in Canada and one study in Turkey. To provide a visual representation of our search and screening process, we have included the PRISMA flow diagram ( Fig. 1 ).

### Overview of the Reviewed Studies' Characteristics

A total of 804 participants with 1,069 facial injuries were evaluated in the reviewed studies. Among these participants, 50.1% ( *n* = 403) received absorbable sutures, while 49.9% ( *n* = 401) received nonabsorbable sutures. Table 1 provides an overview of the characteristics of the sutures used in the studies. The review included a total of nine papers, with four of them focusing exclusively on pediatric cases and one specifically examining cosmetic procedures (septorhinoplasty) in adults. Two papers discussed the cosmetic outcomes of wound closures on superficial and cutaneous skin, with one paper specifically focusing on facial skin wounds. One study assessed facial scar healing using photographic evidence, while another examined the cosmetic outcomes of facial lacerations repaired in an emergency department. Table 2 presents the characteristics of the included studies. During the selection process, eight papers were excluded as they were of the wrong study type. These included four systematic reviews, three non-RCTs, and one case report. Additionally, 10 papers were excluded due to investigating the wrong intervention. These studies explored various comparisons such as different types of wound closures (e.g., Steri-strips or glue with sutures vs. sutures alone) in facial laceration closures, single-layer versus double-layer closure in facial lacerations, sutures used in body contouring, V-Y advancement flaps repairs for facial defects, and sliding knots in monofilament and multifilament sutures. Four papers were excluded as duplicates found in other databases. Furthermore, one paper was excluded as it did not focus on facial wounds but rather abdominal wounds. Another paper was excluded due to incorrect publication year (1986). Additionally, one study was excluded as it had been retracted after publication. Finally, one study was excluded as it examined the wrong outcomes.

**Table 1:** The types of sutures used in the trials included

Study	Suture type		Subcutaneous sutures	Suture length (in cm)
	Absorbable suture	Nonabsorbable suture		
Parell and Becker 16	5–0 vicryl rapide	5.0 prolene	Yes (absorbable suture—poliglecaprone 25)	N/A
Karounis et al 17	4.0–5.0 plain catgut	4.0, 5.0, 6.0 nylon	N/A	N/A
Holger et al 18	6.0 rapid absorbing catgut	6.0 nylon	N/A	N/A
Rosenzweig et al 19	5.0 poliglecaprone-25	6.0 prolene	Yes (5–0 poliglecaprone-25)	3.66
Luck et al 20	5.0 fast absorbing gut	5.0 nylon	Yes (fast-absorbing catgut)	N/A
Eisen et al 21	5.0 fast absorbing gut	5.0 polypropylene	Yes (polyglactin 910)	N/A
Erol et al 22	6/0 polyglytone 6211	6/0 Polypropylene	N/A	N/A
Moran et al 23	5–0 polyglactin 910 (vicryl rapide)	5–0 nylon (Ethilon)	N/A	N/A
Luck et al 24	5.0–6.0 fast absorbing surgical gut	5.0–6.0 nylon	N/A	N/A

**Table 2:** Characteristics of the included studies

Study	Title	Country	Population	Age		Gender (M/F)	Sample size		Follow-up duration	Type of operation	Wound length (in cm)		Inclusion criteria
				Absorbable	Nonabsorbable		Absorbable	Nonabsorbable			Average	Range	
Parell and Becker (2003) 16	Comparison of absorbable with nonabsorbable sutures in closure of facial skin wounds	United States	Adult patients	≥18 years old	≥18 years old	Not specified	44	44	Regular intervals for 6 months	Facial skin cancers removal	7.5	3.5–12	Not mentioned
Karounis et al (2004) 17	A randomized, controlled trial comparing long-term cosmetic outcomes of traumatic pediatric lacerations repaired with absorbable plain gut versus nonabsorbable nylon sutures	Canada	1–18 years old	8.1 years old	9.5 years old	58/37	50	45	Short/Long term	Traumatic lacerations: face, torso, and extremities	2	Not mentioned	Enrolled all pediatric patients under the age of 18 who arrived at the emergency department with lacerations that were less than 12 hours old and required sutures
Holger et al (2004) 18	Cosmetic outcomes of facial lacerations repaired with tissue-adhesive, absorbable, and nonabsorbable sutures	United States	≥5 years old	27.9	30.2	69/27	28	29	9–12 months	Facial lacerations	24.7	10–24.7	Consecutive patients who sought medical attention during the presence of physician assistants from 7:00 a.m. to 2:00 a.m. daily, patients with facial lacerations who were 5 years and older, and patients who could provide consent (assent and parental consent for ages 5–17) and agreed to attend follow-up examinations
Luck et al (2008) 24	Cosmetic outcomes of absorbable versus nonabsorbable sutures in pediatric facial lacerations	United States	1–18 years old	Not mentioned	Not mentioned	35/12	23	24	5–7 days and subsequently 3 months	Facial lacerations	1.9	1–3	The study included healthy patients aged 1 to 18 years with facial lacerations ranging from 1 to 5 cm in size
Rosenzweig et al (2010) 19	Equal cosmetic outcomes with 5–0 poliglecaprone-25 versus 6–0 polypropylene for superficial closures	United States	Adult patients	Not mentioned	Not mentioned	Not specified	48	48	Week 1 and after 4 months	Facial wounds resulting from Mohs microscopic surgery	3.66	Not mentioned	Patients with facial Mohs surgery defects (excluding periorbital and nasal defects) Defects appropriate for primary linear complex closure Defects longer than 2 cm and reaching the depth of the subcutis or deeper
Luck R et al. (2013) 20	Comparison of cosmetic outcomes of absorbable versus nonabsorbable sutures in pediatric facial lacerations	United States	1–18 years old	6.9	5.5	53/45	49	49	Between days 4 and 7 and between 3 and 4 months	Facial lacerations (linear)	Not mentioned	Not mentioned	Not mentioned
Eisen et al (2020) 21	Cosmetic outcomes with the use of 5–0 Polypropylene versus 5–0 fast absorbing plain gut for cutaneous wound closure: a randomized evaluator blind trial	United States	≥18 years	64.3	Not mentioned	31/19	50	50	3 months	Lacerations: head and neck, torso, and extremities	5.8	Not mentioned	Age 18 years or older Able to provide informed consent Scheduled for a cutaneous surgical procedure with predicted linear closure Willingness to attend follow-up visits No restrictions on body site
Erol et al (2020) 22	Comparison of rapid absorbable sutures with nonabsorbable sutures in closing transcolumellar incision in septorhinoplasty: short-term outcomes	Turkey	Adult patients, 19–57 years	31.5	33.1	25/39	32	32	3 months and 1 year	Open rhinoplasty	Not mentioned	Not mentioned	Patients who underwent primary open rhinoplasty between May 2017 and February 2018 Patients who provided written informed consent Patients who underwent a complete otorhinolaryngological examination, including evaluation of the nasal cavity and nasopharynx through anterior rhinoscopy and flexible endoscopy Patients without a history of previous nasal surgery (aesthetic or tumor) or systemic diseases affecting connective tissues Patients with no complaints of hypertrophic scars or keloids on the skin
Moran et al (2020) 23	Photographic assessment of postsurgical facial scars epidermally sutured with rapidly absorbable polyglactin 910 or nylon: a randomized clinical trial	Canada	≥18 years	70.6	Not mentioned	51/49	105	105	1 week, 2 and 6 months	Facial wounds resulting from Mohs microscopic surgery	7.23	4–18.9	Age 18 years or older, regardless of immune status Patients with a facial post-Mohs micrographic surgery defect requiring a wound repair that is at least 4 cm in length

### Analyzing the Primary Outcomes of the Data

#### Visual Analog Cosmesis Scale

**Fig. 2:**
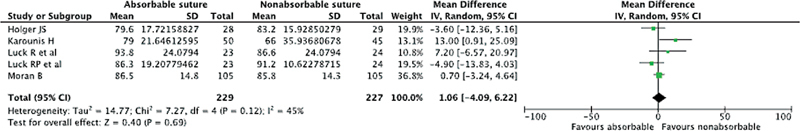
The Forest plot for visual analog cosmesis scale following the use of absorbable suture and nonabsorbable suture for facial wound skin closure. CI, confidence interval; MD, mean difference.

Out of the reviewed studies, only four specifically evaluated cosmetic wounds using the VAS assessment scale. In all four studies, patients were assessed using a 100-point VAS. The group using absorbable sutures included a total of 229 patients, while the group using nonabsorbable sutures included 227 patients. Regarding the comparison between the two groups, the mean difference (MD) in VAS scores was 1.06 (95% CI −4.06, 6.22; *p* = 0.12; *I* ^2^ = 45%). Additionally, the standardized MD was 0.08 (95% CI −0.18, 0.33; *p* = 0.16; *I* ^2^ = 39%). Fig. 2 displays these results.

#### Infection Rate

**Fig. 3:**
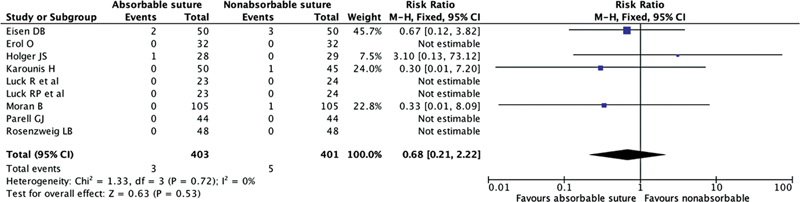
The Forest plot for surgical site infection following the use of absorbable suture and nonabsorbable suture for facial wound skin closure. CI, confidence interval.

All nine studies included in the review reported infection rates, as shown in Fig. 3 . However, five of these studies did not provide specific information regarding wound infections. Among the patients treated with absorbable sutures ( *n* = 403), three cases of wound infections were identified. In the group treated with nonabsorbable sutures ( *n* = 401), four cases of wound infections were identified. The overall RR of wound infections was 0.68 (95% CI 0.21; 2.22; *p* = 0.52; *I* ^2^ = 0%).

#### Wound Dehiscence

**Fig. 4:**
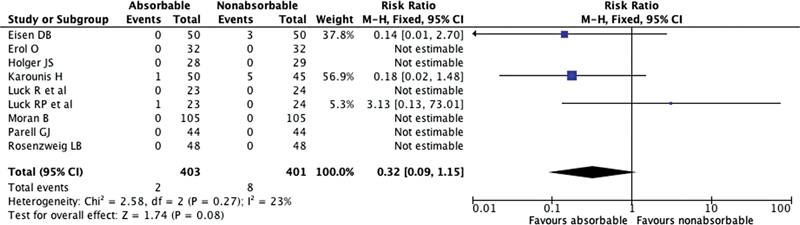
The Forest plot for wound dehiscence following the use of absorbable suture and nonabsorbable suture for facial wound skin closure. CI, confidence interval.

Out of the nine reviewed studies, four studies reported wound dehiscence rates, as depicted in Fig. 4 . According to these four studies, two cases of wound dehiscence were observed in the group treated with absorbable sutures, while eight cases were observed in the group treated with nonabsorbable sutures. The overall relative risk (RR) of wound dehiscence was 0.32 (95% CI 0.09, 1.15; *p* = 0.08; *I* ^2^ = 23%).

#### Scar Hypertrophy, Erythema/Inflammation, and Stitch Marks

**Fig. 5:**
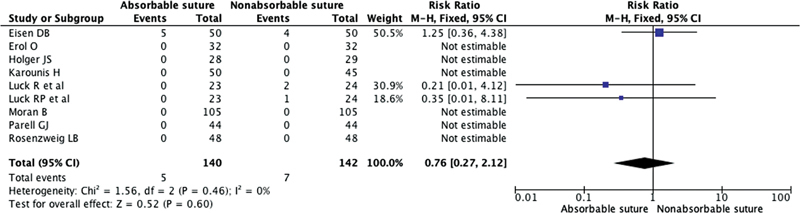
The Forest plot for scarring following the use of absorbable suture and nonabsorbable suture for facial wound skin closure. CI, confidence interval.

Three studies included in the review reported scar-related complications, specifically hypertrophy or keloids. Among these three studies, five cases of scar-related complications were observed in the group treated with absorbable sutures, while seven cases were observed in the group treated with nonabsorbable sutures. In the combined analysis, the RR of scar-related complications was 0.76 (95% CI 0.27, 2.12; *p* = 0.6; *I* ^2^ = 0%) as shown in Fig. 5 .

In one study conducted by Parell and Becker (2003), only one patient in each group experienced erythema/inflammation as a complication. Regarding stitch marks, as mentioned in the study by Parell and Becker (2003), patients treated with nonabsorbable sutures ( *n* = 4) had a higher incidence of stitch marks compared with patients treated with absorbable sutures ( *n* = 2). 16 The risk ratio (RR) of stitch marks was 0.67 (95% CI 0.12, 3.80).

### Risk of Bias

**Fig. 6:**
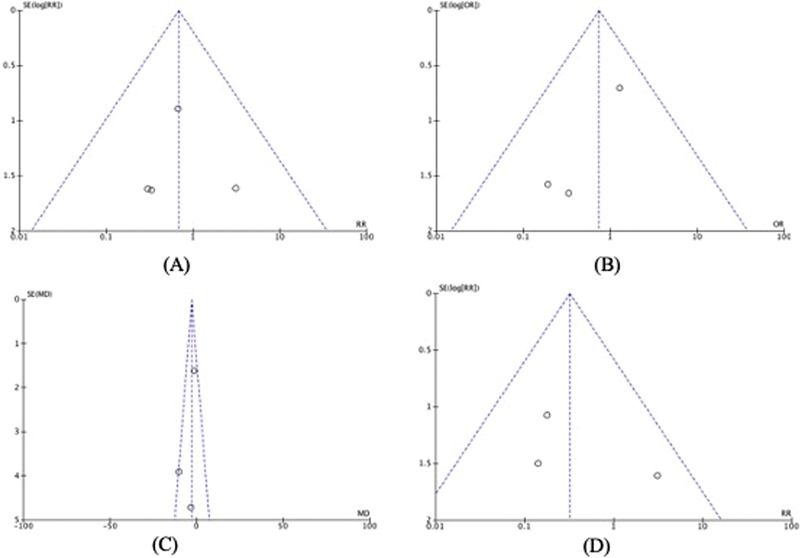
Funnel plots assessing publication bias for various outcomes: ( **A** ) infection, ( **B** ) scarring, ( **C** ) visual analog scale, and ( **D** ) wound dehiscence. The plots demonstrate a symmetrical distribution of studies, indicating no significant publication bias in this meta-analysis.

The assessment of bias risk was conducted by two reviewers simultaneously and independently. The Cochrane Risk of Bias Assessment Tool for Randomized Trials was used to assess the risk of bias for eligible RCTs. The assessment was performed using the Revised Cochrane tool, as shown in Table 3 . 14 Among the included RCTs, eight were considered to have a low risk of bias, while only one was considered to have a high risk of bias based on the Revised Cochrane tool. In terms of the level of evidence and grading recommendations from the American Society of Plastic Surgery, all of the included articles were categorized as level I evidence, as indicated in Table 1 . 15 Publication bias was assessed using a funnel plot, as shown in Fig. 6 . The funnel plot analysis indicated no significant evidence of publication bias in this meta-analysis. The funnel plots appeared largely symmetrical, suggesting that there was no substantial bias.

**Table 3:** Review authors' judgments about each risk of bias item for each included study

Study	Bias arising from the randomization process	Bias due to deviations from intended interventions	Bias due to missing outcome data	Bias in measurement of the outcome	Bias in selection of the reported result	Overall RoB
Parell and Becker (2003) 16	Low	Low	Low	Low	Low	Low
Karounis et al (2004) 17	Low	Low	Low	Low	Low	Low
Holger et al (2004) 18	Low	Low	Low	Low	Low	Low
Rosenzweig et al (2010) 19	Low	Low	Low	Low	Low	Low
Luck et al (2013) 20	Low	Low	High	Low	Low	High
Eisen et al (2020) 21	Low	Low	Low	Low	Low	Low
Erol et al (2020) 22	Low	Low	Low	Low	Low	Low
Moran et al (2020) 23	Low	Low	Low	Low	Low	Low
Luck et al (2008) 24	Low	Low	Low	Low	Low	Low

Abbreviation: RoB, Cochrane risk of bias assessment tool for randomized trials.

## Discussion

This systematic review and meta-analysis of RCTs extensively reviewed a large number of papers totaling 28,411. From this comprehensive search, nine articles were deemed eligible for inclusion in the review. The evaluation focused on facial injuries in a total of 804 patients, with 50.1% of them receiving absorbable sutures and the remaining patients treated with nonabsorbable sutures. The analysis of various outcomes such as VACS, infection, and wound healing did not reveal any statistically significant differences between absorbable and nonabsorbable sutures. However, there was a statistically significant finding in terms of the VACS reported by patients, favoring absorbable sutures ( *p* = 0.01).

Based on this systematic review, the authors examined the evidence comparing absorbable and nonabsorbable sutures for the closure of facial wounds. Based on our findings, there were no significant differences between absorbable and nonabsorbable suture materials in terms of VACS, VAS, infection, dehiscence, hypertrophy, erythema, or suture marks. However, a majority of authors preferred absorbable sutures (50.1%). It is important to note that there is a lack of consistency in cosmetic assessment methods and a poor quality of evidence supporting this preference. Previous RCTs and comparative studies have reported similar conclusions. 7 8 25 26 These studies have compared the effects of absorbable and nonabsorbable skin closure sutures on the face. Absorbable sutures also showed favorable outcomes in terms of postoperative complications and surgical site infection. However, due to a lack of trials and a small number of patients, a comparison between continuous and interrupted stitch closure of the skin could not be made in this review. One of the main strengths of this study is the review of the highest level of evidence available. The review also followed a strict research protocol, as described in the “Methods and Materials” section. Additionally, the researchers involved in this study conducted the search process independently. In the study by Erol et al (2020), it is noted that the columella incision size was relatively small. 22 This characteristic of the study sample may have implications for the outcomes and findings related to facial skin closure techniques. While the small columella incision size in this particular study may limit the generalizability of its findings to cases with larger incisions, it is important to consider the potential impact of incision size on the overall outcomes of facial skin closure. Further research is warranted to explore the influence of columella incision size on the effectiveness of absorbable versus nonabsorbable sutures.

This review has certain limitations that should be considered. First, the inclusion of different population groups may have led to variations in outcomes related to cosmetic satisfaction. Additionally, the use of various types of suture materials across the studies introduces another source of heterogeneity. Furthermore, there was inconsistency in the duration of follow-up among the included papers. The heterogeneity in outcomes represents a significant limitation, despite the shared primary outcome of assessing cosmetic results. Furthermore, our systematic review and meta-analysis included a total of nine articles, which may be considered a small sample size. While we aimed to include high-quality studies with relevant data on absorbable versus nonabsorbable sutures for facial skin closure, the limited number of studies may affect the generalizability of our findings. The small sample size restricts the statistical power and may limit the precision of the estimated effect sizes. Therefore, caution should be exercised when interpreting the results, and further research with larger sample sizes is needed to enhance the robustness of the evidence.

It is necessary to use reliable and consistent measures to assess outcomes generally. Currently, there is no evidence suggesting that one assessment tool is superior to others. However, in recent years, photographic assessment has gained significance as it enables accurate documentation of scars and objective evaluation of wounds over time. The choice of skin closure methods has an impact on both patient outcomes and health care resources. To better understand the effectiveness of absorbable versus nonabsorbable sutures in facial injuries, further well-designed research is needed. It is crucial to conduct studies with follow-up periods of at least 1 year to assess outcomes such as scar formation, wound complications, and cosmesis. It is worth noting that the removal of nonabsorbable sutures in pediatric patients can be distressing and often requires the use of general anesthesia. 10

## Conclusion

In conclusion, the postoperative outcomes of facial skin closure using absorbable or nonabsorbable sutures showed no significant differences in terms of clinical, aesthetic, or patient-reported outcomes. Absorbable suture material can be considered an effective alternative to nonabsorbable sutures for closing facial wounds. However, further RCTs are needed to assess the long-term outcomes of both types of sutures. There is a clear need for more well-designed studies focusing on surgical wound closure following facial injuries, particularly with controlled sutures and standardized criteria for evaluating outcomes.
